# Harnessing the Medicaid Analytic eXtract (MAX) to Evaluate Medications in Pregnancy: Design Considerations

**DOI:** 10.1371/journal.pone.0067405

**Published:** 2013-06-26

**Authors:** Kristin Palmsten, Krista F. Huybrechts, Helen Mogun, Mary K. Kowal, Paige L. Williams, Karin B. Michels, Soko Setoguchi, Sonia Hernández-Díaz

**Affiliations:** 1 Department of Epidemiology, Harvard School of Public Health, Boston, Massachusetts, United States of America; 2 Division of Pharmacoepidemiology and Pharmacoeconomics, Department of Medicine, Brigham and Women's Hospital and Harvard Medical School, Boston, Massachusetts, United States of America; 3 Department of Biostatistics, Harvard School of Public Health, Boston, Massachusetts, United States of America; 4 Obstetrics and Gynecology Epidemiology Center, Department of Obstetrics, Gynecology, and Reproductive Biology, Brigham and Women’s Hospital, Harvard Medical School, Boston, Massachusetts, United States of America; 5 Institute for Prevention and Cancer Epidemiology, University of Freiburg Medical Center, Freiburg, Germany; 6 Duke Clinical Research Institute, Duke University School of Medicine, Durham, North Carolina, United States of America; The Ohio State Unversity, United States of America

## Abstract

**Background:**

In the absence of clinical trial data, large post-marketing observational studies are essential to evaluate the safety and effectiveness of medications during pregnancy. We identified a cohort of pregnancies ending in live birth within the 2000–2007 Medicaid Analytic eXtract (MAX). Herein, we provide a blueprint to guide investigators who wish to create similar cohorts from healthcare utilization data and we describe the limitations in detail.

**Methods:**

Among females ages 12–55, we identified pregnancies using delivery-related codes from healthcare utilization claims. We linked women with pregnancies to their offspring by state, Medicaid Case Number (family identifier) and delivery/birth dates. Then we removed inaccurate linkages and duplicate records and implemented cohort eligibility criteria (i.e., continuous and appropriate enrollment type, no private insurance, no restricted benefits) for claim information completeness.

**Results:**

From 13,460,273 deliveries and 22,408,810 child observations, 6,107,572 pregnancies ending in live birth were available after linkage, cleaning, and removal of duplicate records. The percentage of linked deliveries varied greatly by state, from 0 to 96%. The cohort size was reduced to 1,248,875 pregnancies after requiring maternal eligibility criteria throughout pregnancy and to 1,173,280 pregnancies after further applying infant eligibility criteria. Ninety-one percent of women were dispensed at least one medication during pregnancy.

**Conclusions:**

Mother-infant linkage is feasible and yields a large pregnancy cohort, although the size decreases with increasing eligibility requirements. MAX is a useful resource for studying medications in pregnancy and a spectrum of maternal and infant outcomes within the indigent population of women and their infants enrolled in Medicaid. It may also be used to study maternal characteristics, the impact of Medicaid policy, and healthcare utilization during pregnancy. However, careful attention to the limitations of these data is necessary to reduce biases.

## Introduction

In the United States (US), 50–70% of pregnant women use at least one prescription drug during their pregnancy [1]–[2]. Because pregnant women are routinely excluded from randomized controlled trials [3], post-marketing observational studies provide the information on the effectiveness and safety of medications in pregnancy. Moreover, since many pregnancy outcomes of interest are very rare (e.g., specific malformations occur in 1–30 per 10,000 live births [4]), epidemiologic studies of medications in pregnancy require large data sources. In this context, healthcare utilization databases are an important resource for the study of medications [5].

Medicaid is the joint state and federal health insurance program in the US for low-income individuals. States provide Medicaid claims to the Centers for Medicare and Medicaid Services (CMS) through the Medicaid Statistical Information System (MSIS), and Medicaid Analytic eXtract (MAX) data are extracted from the MSIS to support research and policy analysis [6]. MAX data are available through CMS conditional on data use agreements and fees [7]. The data contain beneficiary enrollment and healthcare utilization claims, including outpatient pharmacy dispensing and inpatient and outpatient diagnosis and procedure claims. Healthcare utilization data are collected for the administration of and payment for healthcare services [5] and, consequently, their use for research is not straightforward.

Medicaid covers the medical expenses of over 40% of births in the US [8]. The population of pregnant women enrolled in Medicaid is young, racially diverse, and low-income; this type of population is typically underrepresented in volunteer-based studies and in studies using private health insurance data.

Statewide Medicaid, Canadian province-wide, and health maintenance organization healthcare utilization data have been used to identify woman-infant linked pregnancy cohorts [9]–[17]. One prior study utilized a small cohort of pregnant women from MAX who were not linked to infants [18]. However, no previous studies have identified a woman-infant linked pregnancy cohort from nationwide Medicaid data, which allows for a larger cohort size and permits the study of regional variation in medication and healthcare utilization during pregnancy.

Below we describe the methods used to identify pregnancies in MAX, link women to their live born infants, select a cohort of woman-infant pairs, and reduce the limitations of MAX for studies of medications in pregnancy and other healthcare factors. We also present the characteristics of women in the cohort and the frequency of several pregnancy outcomes.

## Materials and Methods

### Data Source

We obtained MAX data for all states and Washington, DC, except Arizona, which had inaccurate personal identifiers [19]; data for US territories were not available. Data were available from 2000–2007, except for Maine and Tennessee between 2000–2001 because of quality concerns and for Maine between 2005–2007 because only the Prescription Drug (RX) and Personal Summary (PS) files were available [19]–[20]. We utilized the PS file to obtain demographic and enrollment information, the Inpatient (IP) file to identify hospital diagnosis and procedure codes, the Other Therapy (OT) file to identify diagnosis and procedure codes from outpatient hospitals, clinics, and physicians treating beneficiaries outside a hospital or during a hospitalization, and the RX file to identify outpatient pharmacy dispensings [21]. We used the state-assigned MSIS identification number (MSIS_ID) to identify unique individuals [22] and the state-assigned Medicaid Case Number to identify family units [9], [11]. Programming was conducted with SAS software, Version 9.2 for Windows (SAS Institute, Inc., Cary, North Carolina, USA). This project was approved by the Brigham and Women’s Hospital and Harvard School of Public Health Institutional Review Boards and a data use agreement was approved by CMS.

### Identification of Deliveries and Delivery Date Ranges

Financial criteria alone do not qualify individuals for Medicaid; rather, individuals must also belong to an appropriate eligibility group to qualify, namely children under age 21, adults with dependent children, pregnant women, individuals with disabilities, and seniors [23]. We restricted the source population to females 12–55 years old who were enrolled in Medicaid for at least one month between 2000 and 2007 according to the PS file; thus we excluded the small proportion of individuals who were missing eligibility information although they had Medicaid claims [20]. We also excluded individuals whose Case Number was missing, zero or ended in 8 zeros.

To identify inpatient deliveries from the source population, we utilized the MAX delivery code variable, which is only available in the IP file and identifies hospitalizations with a delivery-related *International Classification of Diseases*, Ninth Revision (ICD-9) diagnosis code [24]. We also utilized delivery-related ICD-9 procedure codes from the IP file and *Current Procedural Terminology*, Fourth Edition (CPT-4) codes (Table S1 in File S1) from the OT file that had a service date during a hospitalization. The inpatient delivery date range was the window between the maternal admission and discharge dates associated with the delivery-related codes.

To identify outpatient (i.e., physician, clinic, or outpatient hospital) delivery-related claims, we utilized the delivery procedure codes from the OT file. A large proportion of the outpatient delivery-related procedures were for post-partum care, which could occur several days after delivery. We defined the outpatient delivery date range as the five days before and after the delivery-related procedure. If the date of an outpatient delivery-related procedure overlapped with an inpatient delivery date range for the same woman, then the outpatient delivery-related claim was removed.

**Figure 1:**
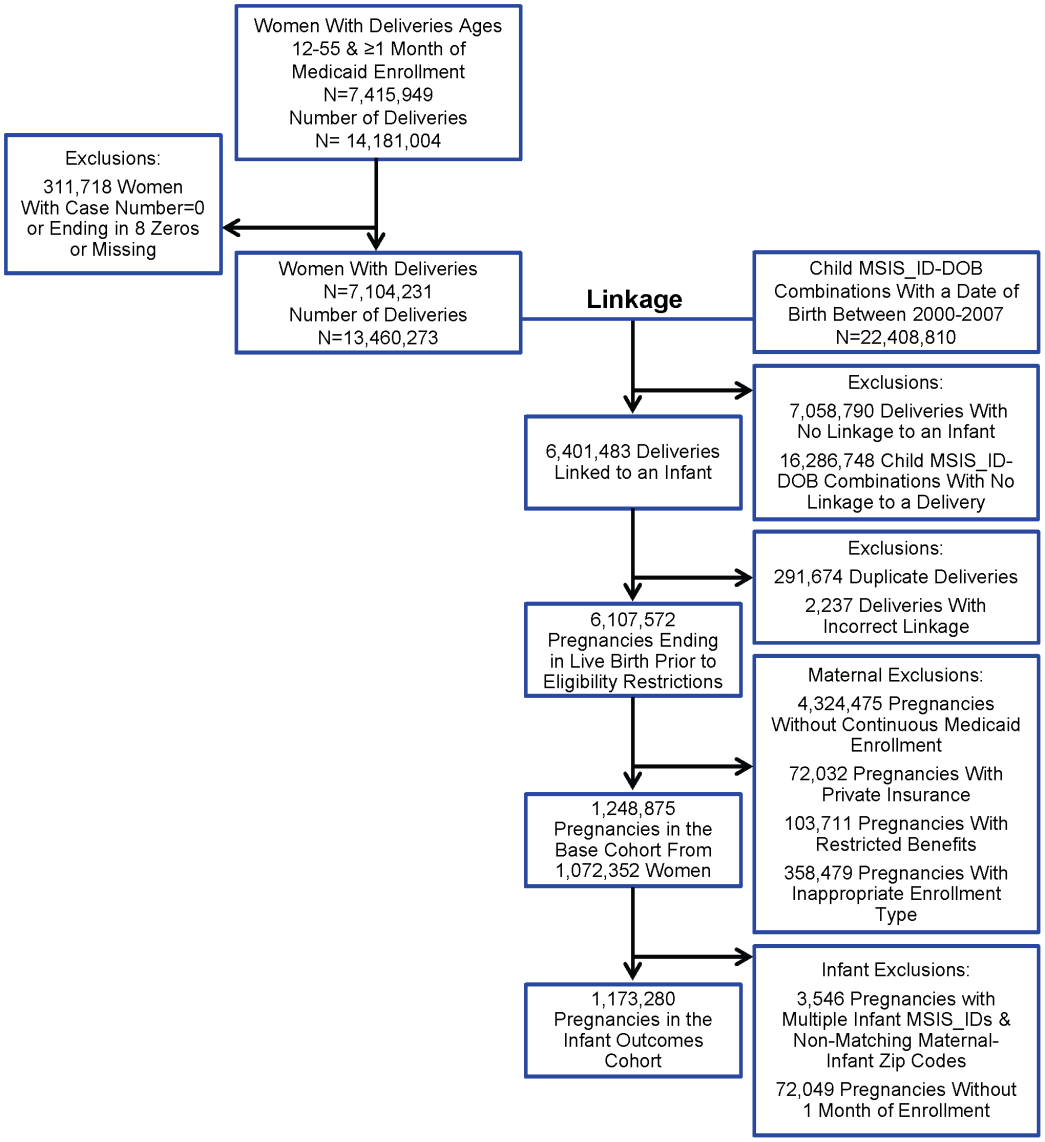
Overview of the linkage and cohort identification process; Medicaid Analytic eXtract, 2000–2007.

A woman could have more than one delivery identified either because she had more than one pregnancy during the study period or because she had the same delivery identified more than once with unique delivery date ranges. Instead of selecting one delivery per woman during a certain time period [9], [25], we retained all deliveries to maximize the yield of the linkage step. Then we removed the duplicate deliveries after linkage. As a result, the linkage proportion that we report will be lower than algorithms that delete duplicate deliveries prior to linkage. We identified 13,460,273 deliveries from 7,104,231 women with valid Medicaid Case Numbers (Figure 1).

### Identification of Children

We identified children with a date of birth (DOB) between 2000–2007 and a Case Number that was not missing or zero and did not end in 8 zeros. Some MSIS_IDs are associated with more than one DOB, e.g., correct DOB, mistyped DOB and DOB incorrectly assigned as the first date of Medicaid eligibility. We consolidated infants with the same Case Number and DOBs less than three days apart after, rather than before, linkage. There were 22,408,810 different MSIS_ID-DOB combinations available for linkage to deliveries.

### Woman-infant Linkage

**Figure 2:**
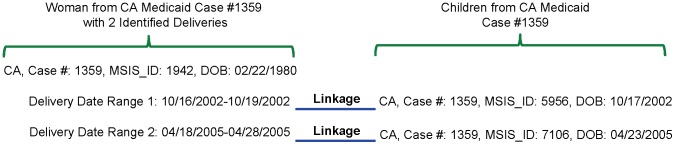
Hypothetical example of woman-infant linkage by state, Case Number, and delivery date range/date of birth. CA, California.

Successful linkage requires the mother to be enrolled in Medicaid on the child’s DOB, accurate recording and consistent use of the Case Number within families, and accurate dates of delivery and birth. Within each state, we linked women to infants by the Case Number. Table S2 (in File S1) describes the elements of the Case Number that were used for linkage in each state. We were unable to identify a matching algorithm for New York between 2000–2003 and for Connecticut and Montana in all study years. We also required that the infant’s DOB fell within the woman’s delivery date range to link the correct siblings to each delivery (Figure 2).

First, within each state, we linked inpatient deliveries with the pool of child MSIS_ID-DOB combinations, and then we linked outpatient delivery-related claims with child MSIS_ID-DOBs that had not been linked. The delivery linkage percentage was defined as the proportion of delivery date ranges (there could be more than one for the same delivery) that linked to a MSIS_ID-DOB combination (there could be more than one per infant). The child linkage percentage was defined as the proportion of child MSIS_ID-DOB combinations that linked to a delivery. While generally informative, these linkage percentages should be interpreted with caution. The same delivery could be counted in the denominator of the delivery linkage percentage more than once if more than one delivery date range was identified, and the same child could be counted in the denominator of the child linkage percentage more than once if the child had more than one MSIS_ID or DOB recorded. Likewise, linked deliveries and children could be counted in the numerators of both percentages more than once if the linked child had more than one MSIS_ID. Finally, the child linkage percentages are low because not all mothers of children from the pool were enrolled in Medicaid on their child’s DOB, making their deliveries unavailable for linkage. Besides matching women with their offspring, the linkage procedure contributed to the de-duplication of infants and the establishment of delivery date, as explained below.

### Post-linkage Cleaning

To produce a cohort of unique pregnancies from the linked deliveries, we implemented several data cleaning steps to remove deliveries that were incorrectly linked or duplicated. To remove incorrectly linked deliveries, we excluded all infants that were linked to more than one woman’s MSIS_ID. Then we removed all deliveries that were linked to infant MSIS_IDs with DOBs more than two days apart (more than three days apart for outpatient deliveries) and less than 24 weeks apart. This step preserved multifetal deliveries, but it removed deliveries that were unreasonably close in time. To remove duplicate deliveries, we combined linked deliveries from the same woman into one delivery if the infants’ DOBs were less than three days apart (less than four days for outpatient deliveries). For these deliveries, the earliest DOB was selected as the infants’ DOB and the woman’s delivery date.

### Estimation of the Last Menstrual Period (LMP)

The date of the LMP was estimated because neither gestational length nor the LMP is available in healthcare utilization data. It was assigned to be 245 days before the infant’s DOB for pregnancies that had maternal or infant ICD-9 codes indicative of preterm delivery (644.0, 644.2, and 765.x) and to be 270 days before the infant’s DOB for all other pregnancies [26].

### Women’s Eligibility Criteria

MAX may contain an incomplete record of healthcare claims for the linked women when they are not enrolled in Medicaid, have supplemental private insurance, have restricted benefits, such as pregnancy-related services or prescription drug benefits only, or are enrolled in certain managed care plans [27]. Medicaid beneficiaries can be enrolled in two major types of managed care plans, capitated (i.e., risk-based) or fee-for-service primary care case management (FFS PCCM), or they may not be enrolled in a managed care plan (women not enrolled in these plans are referred to as traditional beneficiaries) [28]. Encounter records, which take the place of claims for services provided to patients enrolled in capitated managed care plans, are incomplete in certain states [29]–[30]. We implemented eligibility criteria based on these Medicaid program provisions and arrangements to increase the completeness of claim information among women included in the cohort.

**Figure 3:**
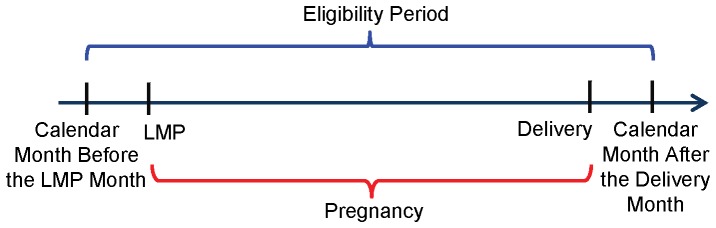
Schematic of the primary eligibility period.

Women were excluded if any of the following four eligibility criteria were not met, according to the PS file, in at least one month during the eligibility period of interest: 1) Continuous enrollment throughout the eligibility period, defined as at least 28 days of enrollment per calendar month. 2) No private insurance. 3) No restricted benefits. 4) Appropriate enrollment type (i.e., capitated managed care, FFS PCCM managed care, or no managed care) depending on state (Table S2 in File S1). We excluded women enrolled in capitated plans in states where they had fewer claims compared to FFS PCCM or traditional beneficiaries. Also, we excluded traditional beneficiaries in states that had a high proportion of women with restricted benefits and in which traditional beneficiaries had few claims; these women likely had unidentified restricted benefits given the high proportion of women with restricted benefits in these states. All pregnancies were excluded in Michigan because the number of claims among all enrollee types was implausibly low. We defined the primary eligibility period (Figure 3) as the calendar month before the LMP month until the calendar month after the delivery month or date of death, whichever occurred first, to ensure follow-up throughout pregnancy. We report the cohort size for the primary eligibility period (i.e., the base cohort) and for shorter and longer eligibility periods, which may also be of interest for certain research questions.

### Multiple MSIS_IDs and Infants’ Eligibility Criteria

MAX anomalies tables indicate that individuals may receive more than one MSIS_ID within the same state [20]. Although it is legitimate for multifetal pregnancies to have more than one infant MSIS_ID, in at least some pregnancies with multiple infant MSIS_IDs, infants may have been assigned a temporary MSIS_ID at birth and later received a permanent MSIS_ID [20].

We required additional infant eligibility criteria for studies of infant outcomes. Because we could not rule out multiple infant MSIS_IDs per pregnancy as an indication of poor linkage quality, we required more stringent eligibility criteria in those pregnancies: if infant zip code was different from maternal zip code, then the infant was excluded. Prior to applying infant eligibility criteria, zip codes did not match in 5.5% of pregnancies linked to one infant MSIS_ID, 25.7% of pregnancies linked to two MSIS_IDs, and 48.7% of pregnancies linked to three MSIS_IDs.

Because multiple infant MSIS_IDs that linked to the same pregnancy may represent the same infant, we pooled eligibility information from all infant MSIS_IDs associated with a pregnancy and required that at least one infant MSIS_ID had either Medicaid enrollment in the month after the birth month or a claim in the month after birth. Pregnancies with neonatal death remained eligible even if they did not meet the enrollment criteria.

### Medication dispensings and Outcome Assessment

Pharmacy claim dates during pregnancy were used to identify pregnant women who were dispensed a medication. Both inpatient and outpatient ICD-9 diagnostic codes were used to identify pregnancy outcomes.

## Results

### Woman-infant Linkage

Overall, of the 10,058,005 identified inpatient deliveries, 55.6% linked to at least one infant, and of the 3,402,268 outpatient (i.e., physician, clinic or outpatient hospital) delivery claims, 23.8% linked to at least one infant. The delivery linkage percentages varied greatly by state (Table 1). From the pool of 22,408,810 child MSIS_ID-DOB combinations, 6.8% would not have met the maternal eligibility criteria applied later because the child’s DOB was within the first 9 months of 2000 or during December 2007 (i.e., data was not available during the maternal eligibility period), and 24.4% linked to an inpatient delivery. Of the remaining 16,457,327 observations that did not link to an inpatient delivery, 3.9% linked to an outpatient delivery.

**Table 1:** The number of deliveries before linkage and the percentage of deliveries that linked to an infant, and the number of child MSIS_ID and date of birth combinations before linkage and the percentage of combinations that linked to a delivery listed by inpatient and outpatient linkage and by state; Medicaid Analytic eXtract, 2000–2007.

	Inpatient Linkage		Outpatient Linkage		Inpatient Linkage		Outpatient Linkage	
State	N Deliveries Before Linkage	% of Deliveries Linked	N Deliveries Before Linkage	% of Deliveries Linked	N Child MSIS_ID-DOBs Before Linkage	% of Child MSIS_ID-DOBs Linked	N Child MSIS_ID-DOBs Before Linkage	% of Child MSIS_ID-DOBs Linked
AK	31553	83.4	7383	41.7	57433	45.9	30541	8.0
AL	335584	83.9	3177	54.0	357467	51.7	164090	1.0
AR	133629	21.7	111902	2.0	272989	10.6	243491	0.7
CA	905520	78.9	417779	38.3	3384571	21.1	2592491	5.4
CO	116192	79.3	12198	47.0	296181	31.1	202696	1.7
CT	20782	0	1970	0	164612	0	164612	0
DC	3927	80.4	915	26.4	51082	6.2	47841	0.3
DE	10149	93.2	23841	70.6	58891	16.1	49230	31.1
FL	543810	71.6	443337	7.1	1483965	26.3	1054754	1.6
GA	415916	35.9	100357	19.1	972752	15.4	812358	1.9
HI	27633	77.6	11758	65.1	74707	28.7	52894	12.9
IA	86326	73.0	21308	55.8	179092	35.2	115181	9.3
ID	61086	74.6	8050	46.8	92037	49.5	45823	5.8
IL	458715	72.3	156915	13.5	931766	35.0	600100	2.4
IN	222255	91.7	178780	18.0	433979	47.0	226903	10.1
KS	80230	90.9	12787	65.1	180418	40.4	105982	6.4
KY	175466	81.1	49526	40.8	336217	42.4	173547	9.0
LA	270481	88.2	138652	12.6	547583	43.6	208107	5.5
MA	81289	89.4	17975	33.4	319082	22.8	243566	1.3
MD	1470560	9.3	13016	10.8	321470	42.5	182008	0.6
ME	15566	93.0	3909	25.6	68627	21.1	53919	1.4
MI	177475	70.7	71736	26.6	626024	20.1	497896	2.9
MN	91875	93.8	70072	67.2	316557	27.2	214314	19.2
MO	253912	54.8	39871	31.2	402150	34.3	261520	3.8
MS	184337	90.2	93213	11.6	353758	47.3	138740	4.8
MT	27084	0	4093	0	52429	0	52429	0
NC	484133	17.3	44800	9.3	679462	12.3	594079	0.5
ND	16679	96.0	3334	36.2	33598	47.7	17335	4.4
NE	25261	79.7	8158	54.6	125239	16.1	104822	3.9
NH	22699	94.0	3519	46.6	55521	38.5	33832	3.4
NJ	89215	84.4	88993	53.4	521386	14.5	428403	9.8
NM	93901	85.4	28986	72.7	222010	36.1	129329	13.8
NV	31258	89.5	11527	75.4	180635	15.5	147048	5.0
NY	642194	52.1	107573	34.3	1396520	23.8	1056220	2.8
OH	252528	94.2	154680	17.1	725433	32.8	481332	2.6
OK	156994	87.9	37478	55.5	392679	35.2	243912	7.2
OR	98716	88.0	23427	60.2	235288	36.9	146705	7.8
PA	111772	93.0	17485	31.2	634621	16.3	529191	0.8
RI	46047	91.1	8280	31.4	63926	48.0	32622	4.8
SC	199463	16.1	116129	1.7	341982	9.4	309065	0.5
SD	30083	93.8	3170	50.1	59627	47.4	30916	4.0
TN	168714	80.0	71995	69.3	447117	30.2	309663	14.1
TX	831729	9.1	486918	1.0	2349734	3.2	2272382	0.1
UT	51991	95.7	9264	81.6	203126	24.5	152506	2.3
VA	151876	87.1	62204	58.6	391259	33.9	255155	12.2
VT	18386	91.9	2807	56.8	38560	43.9	20994	6.1
WA	117179	84.4	52704	63.9	427660	23.1	327281	8.8
WI	154097	94.0	33966	36.9	354202	40.9	174700	5.2
WV	40739	85.8	6237	47.5	149386	23.5	101601	2.1
WY	20999	93.9	4114	39.4	44000	45.0	23201	5.2
**Total**	**10058005**	**55.6**	**3402268**	**23.8**	**22408810**	**24.4**	**16457327**	**3.9**

### Post-linkage Cleaning

From the 6,401,483 linked deliveries, 293,911 (4.6%) were removed in the post-linkage cleaning phase, resulting in the identification of 6,107,572 unique pregnancies ending in live birth. Of deliveries that were removed, 99.2% were combined with other deliveries because they were duplicates and the rest appeared to be incorrect linkages.

### Eligibility Criteria

**Figure 4:**
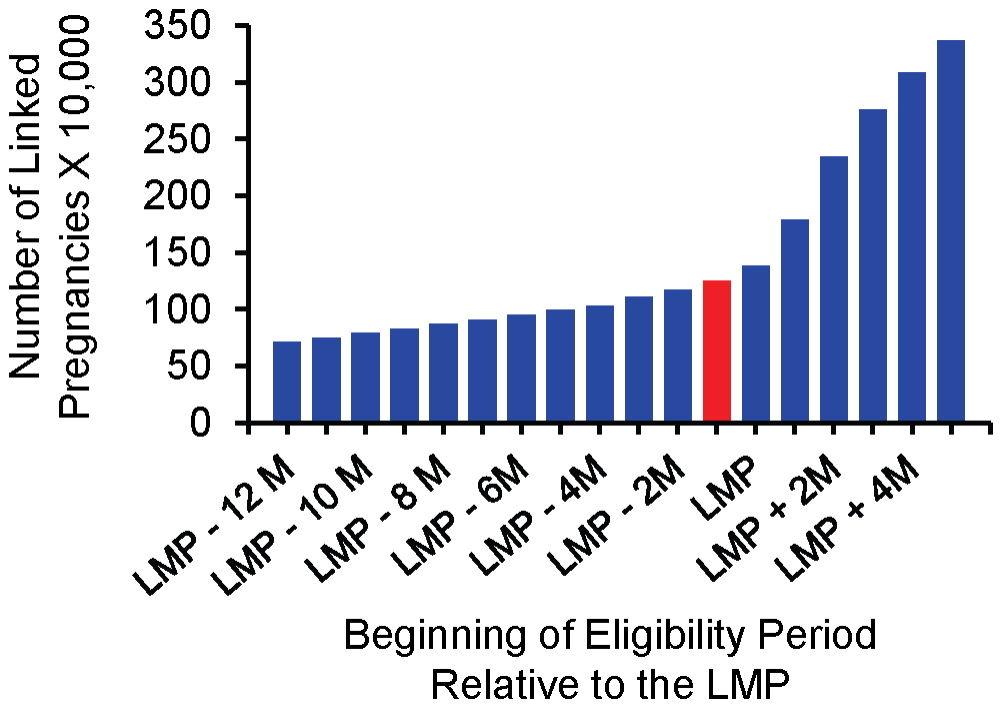
Cohort size by eligibility period; Medicaid Analytic eXtract, 2000–2007. The number of pregnancies in the base cohort (eligible from at least 1 month before the LMP month until the month after the delivery month) is represented in red and the number of pregnancies when additional or fewer months of eligibility are required is represented in blue. The lengths of the eligibility periods decrease when moving away from the vertical axis along the horizontal axis. – indicates the number of months before the LMP and+indicates the number of months after the LMP at which the eligibility period begins, and all eligibility periods continue until the month after the delivery month. LMP, last menstrual period; M, months.

Of the eligibility criteria, the requirement of enrollment throughout pregnancy had the greatest impact on the cohort size (Figure 1). After all the eligibility criteria were implemented, the base cohort consisted of 1,248,875 pregnancies from 1,072,352 women. The cohort size decreased to 633,553 pregnancies when we required that women not be enrolled in capitated managed care plans. The cohort size was sensitive to changes in the maternal eligibility period (Figure 4), and it increased with decreasing eligibility period length requirements. The size of the base cohort was reduced when infant eligibility criteria were implemented too; 1,173,280 (93.9%) pregnancies were available when one month of infant eligibility was required (Figure 1).

### Cohort Description

**Figure 5:**
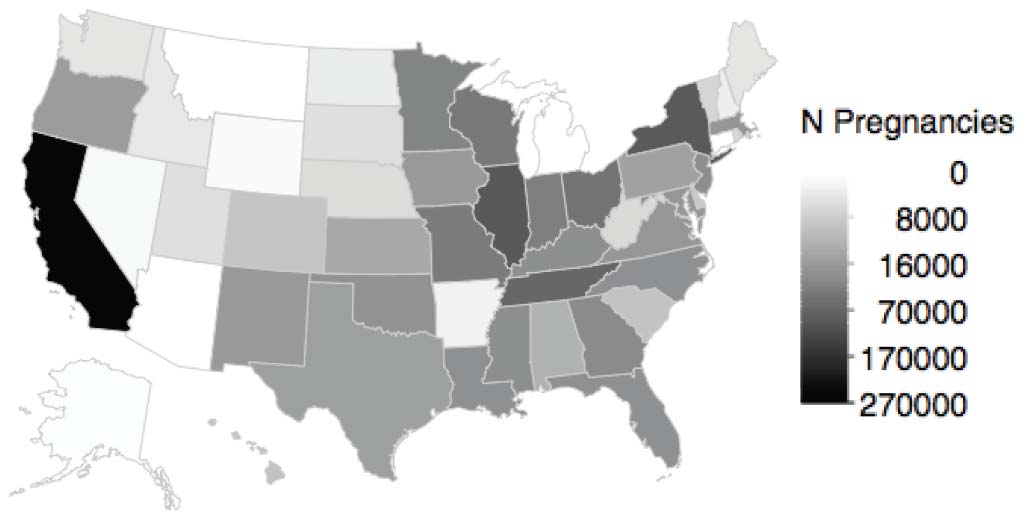
Number of pregnancies contributed to the base cohort by state; Medicaid Analytic eXtract, 2000–2007.

**Figure 6:**
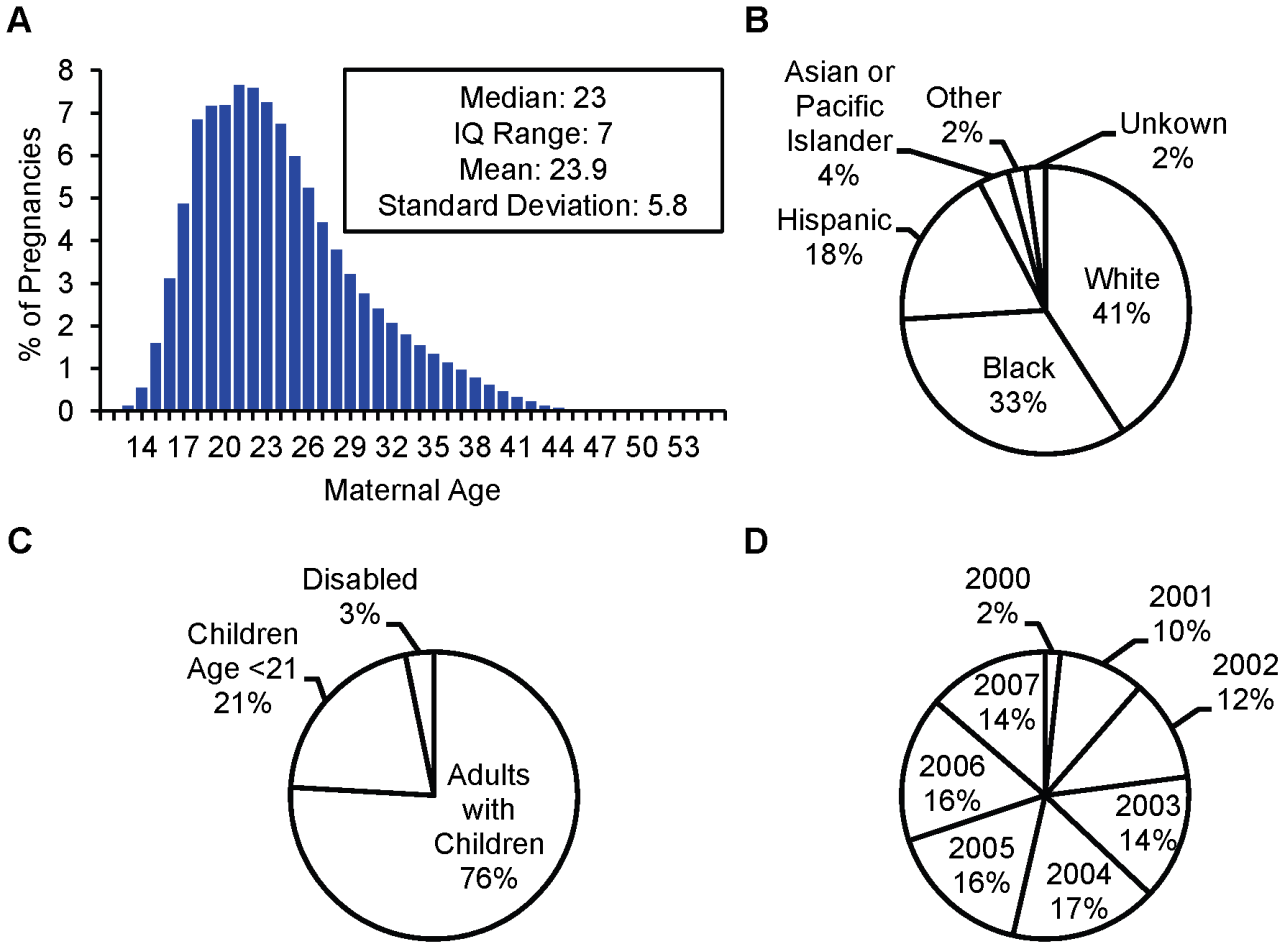
The distribution of maternal characteristics in the base cohort; Medicaid Analytic eXtract, 2000–2007. A) Age, B) Race, C) Medicaid Eligibility Group, and D) Delivery Year.

Figure 5 summarizes the number of pregnancies contributed to the cohort from each state. The largest contribution was from California with 257,148 pregnancies, and the smallest contribution was from Washington, DC, with 533 pregnancies (Table S2 in File S1); 50% of pregnancies were from six states (California, Illinois, New York, Ohio, Tennessee, Wisconsin). The average maternal age was 23.9 years, and 33% of women were Black and 18% were Hispanic (Figure 6). Although women may belong to more than one Medicaid eligibility group, only one group is recorded in MAX; the largest Medicaid eligibility group in the cohort was adults with dependent children. There are relatively few pregnancies from 2000 because of the pre-delivery eligibility requirement. Compared to pregnancies in the linked cohort before eligibility criteria implementation, women in the base cohort were slightly less likely to be white (40.8% vs. 47.4%), more likely to be eligible due to child (15.7% vs 10.5%) or disability status (3.1% vs. 1.0%) on the delivery date, and had a similar age distribution (Table S3 in File S1). In the infant outcomes cohort, 6% of pregnancies had more than one infant MSIS_ID, and among pregnancies with only one infant MSIS_ID, the percentage of pregnancies in which the mother and infant did not share the same zip code was 4.9% (Table S4 in File S1).

Overall, 91% of women had at least one pharmacy dispensing during pregnancy (Table S2 in File S1). Oregon and South Dakota had a relatively lower proportion of pregnant women with pharmacy dispensings (41.6% and 65.3%). The percentage of pregnancies affected by several outcomes is reported in Figure S1.

## Discussion

We developed the methodology to utilize a promising and previously untapped resource for studies of medication exposure during pregnancy and a broad range of maternal and infant outcomes. Healthcare utilization data offer a number of advantages for studies of medications in pregnancy over pregnancy registry and case-control studies including the availability of large, population-based cohorts in which the study of rare outcomes and important subgroups is feasible, the availability of exposed and reference groups from the same population, prospectively collected information on a range of prescription drugs, information on many maternal and neonatal outcomes, and low study cost compared to de novo data collection [5].

Several characteristics of the cohort have face validity. We found that woman-infant pairs with zip codes that did not match were uncommon in most states, which supports accurate linkage. Zip codes may not match for reasons other than poor linkage; infants may not live with their mothers, or maternal zip code may not be current on the delivery date due to changes of residence. Therefore, we did not require zip codes to match in pregnancies with one infant MSIS_ID. Furthermore, the frequency of several pregnancy outcomes was similar to expectations [31]–[32] and the proportion of pregnancies with cesarean delivery tracked over time with national trends [32]–[39], which further supports the data validity. Although we excluded pregnant women who were not linked to infants or did not meet eligibility criteria, these findings suggest that the cohort may be representative of the broader population.

There were several state-specific Medicaid program and data quality factors that contributed to the large variation in the number of pregnancies in the cohort from each state, including the number of pregnant women covered by Medicaid, availability of MAX for all study years, Medicaid benefit restriction and eligibility policies for enrollees, the completeness of claims, quality of Case Numbers, and reuse of Case Numbers within families. The same Case Number is not necessarily shared by all family members [22]. Consequently, fewer deliveries will be linked to infants in states that do not typically assign the same Case Number to women and their infants. Woman-infant linkage is not necessary for studies of healthcare utilization during pregnancy and maternal outcomes [18], [25], and delivery linkage less than 100% will decrease the cohort size, perhaps unnecessarily for these studies. However, falsely identified deliveries and incorrect delivery date and LMP assignment are more likely when deliveries are not linked to infants.

Investigators planning to work with the cohort should be aware of a number of limitations inherent in the data and strategies to address at least some of these issues. To begin with, we restricted the cohort to live births only, because of the infant-linkage step, so it cannot be used to study spontaneous abortion and stillbirths. There is potential for selection bias if the exposure of interest is associated with spontaneous abortion or stillbirth and there are unmeasured common causes of these outcomes and the outcome of interest [40]–[41]. However, many outcomes of interest (e.g., preeclampsia) are conditional on the fetus surviving at least twenty weeks. As with many studies, this bias would mainly result in an underestimation of fetal outcomes that are associated with abortion. Moreover, the pre-linkage cohort could be used to study stillbirth, although a validation study would be warranted.

The method we used to estimate the LMP accurately classified gestational age within 2 weeks for nearly all term and 75% of preterm pregnancies and was superior to other algorithms in one healthcare utilization database [26]. Gestational age at delivery could be obtained by linking MAX data with birth records, but this is not possible at the national level. MAX data do not contain direct personal identifiers such as names and addresses; however social security numbers may be requested from CMS and could be used for birth record linkage in states where social security numbers are available in vital records. Also, multiparity was estimated from the eligibility group and will be misclassified for some women because only one eligibility group is available in MAX data; e.g., women who are disabled and have previous children may only be identified as being disabled instead of multiparous. Because infants may have more than one MSIS_ID, ICD-9 codes should be used to identify multiple gestations.

Furthermore, incorrect linkage, i.e., false woman-infant pairs, could occur if the same Case Number was shared among different families. To our knowledge, there is no CMS validation report regarding the use of the Case Number within families. Incorrect linkage would likely result in non-differential misclassification of infant outcomes which would tend to bias associations towards the null and is problematic for drug safety studies. Social security numbers could be used to validate the linkage method in states where they are available in birth records. It is advisable to conduct sensitivity analyses restricted to a subcohort with potentially higher linkage quality when using the cohort to study infant outcomes.

We privileged internal validity over external validity and statistical power when we implemented maternal eligibility criteria. To ensure complete follow-up through pregnancy, 80% of the originally identified pregnancies were removed. DeVoe et al. reported that healthcare services were under-recorded in Medicaid claims data from Oregon compared to electronic health records [42]. To construct a cohort with a comprehensive picture of healthcare services during pregnancy, we applied a number of eligibility criteria that DeVoe et al. did not [43]. Although these restrictions limit generalizability, they are critical for internal validity. The variability in data quality by state forced us to restrict to the states and enrollment types with acceptable quality and completeness. The requirement of enrollment throughout pregnancy is coupled with a limitation: it effectively excluded women who became eligible for Medicaid because of pregnancy. Consequently, we selected a cohort of women who belong to other Medicaid eligibility groups, i.e., those classified as children, multiparae, and women with disabilities. Nevertheless, the distribution of age and race was similar in the restricted and unrestricted linked cohorts. The proportion of pregnancies exposed to specific prescription drugs and the absolute risk of outcomes may not generalize to the entire population of pregnant women enrolled in Medicaid. The generalizability of measures of association to all pregnant women enrolled in Medicaid and to other populations will depend on differences in the distributions of potential effect modifiers across the populations. Given the large cohort size, measures of association should be stratified by potential effect modifiers such as region, age, race, and parity; stratum-specific results should generalize to non-Medicaid populations even if population averages do not. Moreover, studies requiring shorter follow-up time will permit shorter eligibility periods and therefore will have increased size and may have greater generalizability.

Because date of death is under-recorded in MAX [44], it is possible that we have unintentionally excluded women or infants with apparent lack of eligibility whose date of death was missing. Maternal and infant mortality is rare in the US [45]–[46]; we anticipate few pregnancies were excluded due to under-recorded date of death. Mortality could be studied if the data are linked to the recently released 2008 MAX Date of Death Auxiliary File, which contains more accurate and complete date of death information [44].

Given the decentralized nature of Medicaid data and the sheer number of enrollees, it is unlikely that all cohort members’ claims will be captured in MAX even after applying strict eligibility criteria. Sensitivity analyses should be performed that exclude individuals who are least likely to have complete claim information such as women enrolled in capitated managed care plans [29]–[30] and from states that have a relatively low prevalence of various exposures and outcomes.

Although medical record validation studies have been described for Medicaid data among Medicaid and Medicare dually eligible enrollees [47], the feasibility of obtaining medical records for infant outcomes remains unknown. Medical records at birth may not contain social security numbers or may not be released based on social security numbers alone. MAX data contain hospital identifiers but there is no centralized list of contact information for these identifiers [27].

Some limitations of the MAX cohort are common to other pregnancy cohorts assembled from healthcare utilization data, such as the exclusion of pregnancies ending in miscarriage [9]–[18], [25] and the reliance on algorithms to estimate the date of the LMP [11], [14]–[16], [18], [25]. Furthermore, validity of mother-infant linkage by subscriber or family number is not typically reported.

MAX contains a huge nationwide cohort of pregnant women and prospectively collected data, which permits the study of rare medication exposures and outcomes in an otherwise understudied population. However, linkage of women to their infants is not straightforward, enrollment time is limited for many pregnant women in Medicaid because pregnancy qualifies some women for Medicaid [23], restrictive eligibility criteria are necessary to reduce incomplete claim information, gestational timing is not readily available, and measurement error is unavoidable. The limitations of MAX data may be overcome if investigators choose appropriate study questions, employ careful methodology that favors validity over statistical power, and perform sensitivity analyses to evaluate the limitations of the data and the effect of various assumptions. Ideally, MAX data should be linked to birth records to validate the mother-infant linkage and to obtain additional birth information such as gestational age and birth weight. The cohort size and validity could be improved with modifications to MAX by state Medicaid offices and CMS such as providing complete claim information.

Cohort set-up is resource intensive, from requesting and receiving the data from CMS to linkage and implementation of eligibility criteria. However, once the cohort is assembled, it offers an incredible opportunity to efficiently evaluate medication safety during pregnancy as well as maternal characteristics, the impact of Medicaid policy, and regional differences in healthcare utilization during pregnancy.

## Supporting Information

Figure S1 **Cohort outcomes; Medicaid Analytic eXtract, 2000–2007.** A) Percentage of pregnancies affected by preeclampsia, severe preeclampsia, cesarean delivery, and preterm delivery in the base cohort. B) Percentage of pregnancies with cesarean delivery in the MAX cohort and in the United States according to the National Vital Statistics System [32]–[39] by year for age groups <20, 20–24, 25–29.
(TIF)

File S1 **Supporting Information for MAX Pregnancy Cohort.** Table S1: Delivery-related procedure codes used to identify inpatient and outpatient deliveries from the Medicaid Analytic eXtract, 2000-2007. Table S2: Elements of the Case Number used for linkage, enrollment type exclusions, number of pregnancies, percentage of cohort, and percentage of pregnancies with at least one prescription medication dispensed during pregnancy by state; Medicaid Analytic eXtract, 2000–2007. Table S3: Demographic characteristics on the delivery date among women in the base cohort and women in the linked pre-eligibility cohort; Medicaid Analytic eXtract, 2000–2007. Table S4. The total number of pregnancies and the percentage of pregnancies that have more than one infant MSIS_ID from the infant outcomes cohort, and the number of pregnancies with one infant MSIS_ID from the infant outcomes cohort and the percentage of pregnancies in which woman-infant pairs did not share the same zip code among pregnancies with one infant MSIS_ID from the infant outcomes cohort by state; Medicaid Analytic eXtract, 2000-2007.
(PDF)
