# Supplementary figures and images for: Harnessing the Medicaid Analytic eXtract (MAX) to Evaluate Medications in Pregnancy: Design Considerations
Source: PLoS One. 2013 Jun 26;8(6):e67405. doi: 10.1371/journal.pone.0067405 (PMC3693950; doi:10.1371/journal.pone.0067405)

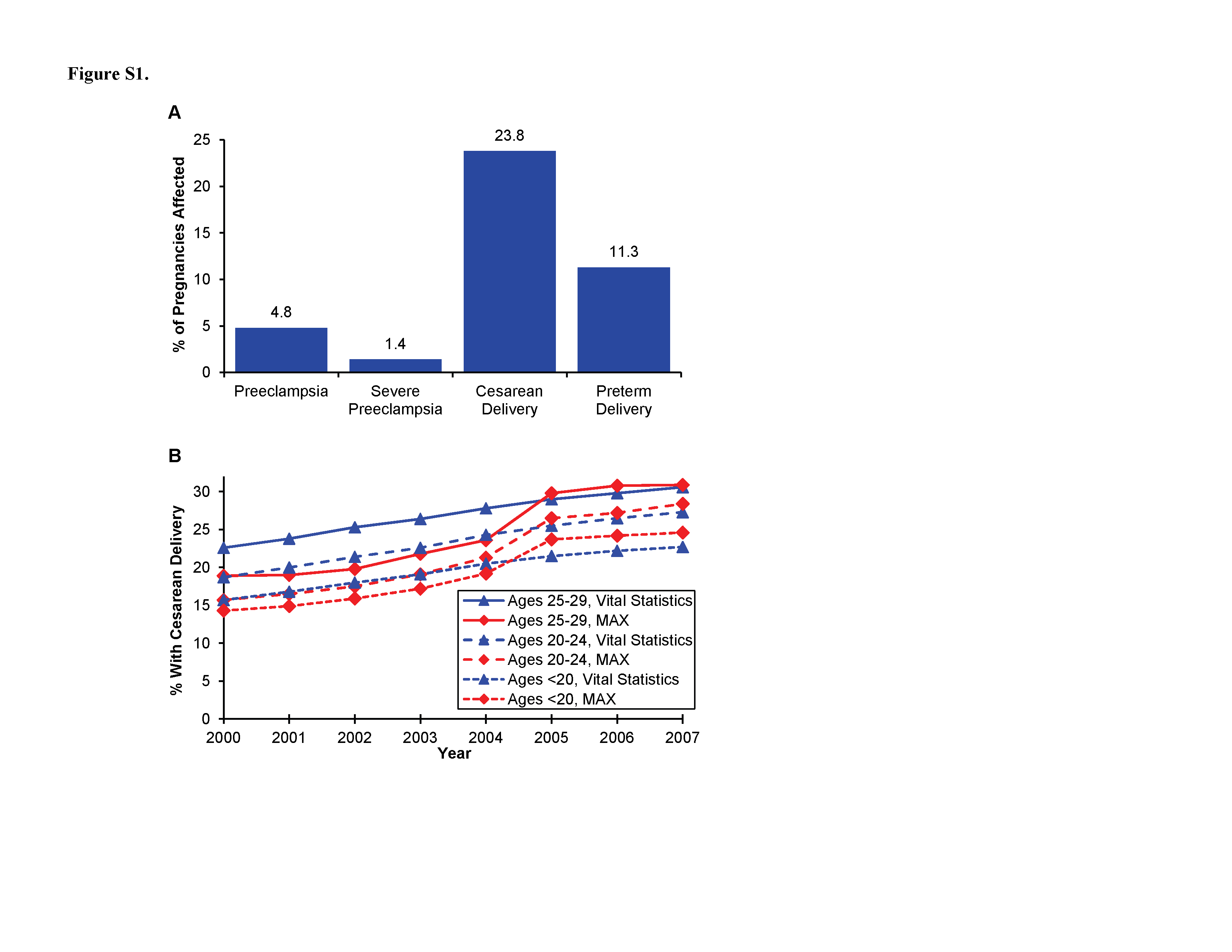

Supplement: Figure S1 — Cohort outcomes; Medicaid Analytic eXtract, 2000–2007. A) Percentage of pregnancies affected by preeclampsia, severe preeclampsia, cesarean delivery, and preterm delivery in the base cohort. B) Percentage of pregnancies with cesarean delivery in the MAX cohort and in the United States according to the National Vital Statistics System [32]–[39] by year for age groups <20, 20–24, 25–29. (TIF) [file pone.0067405.s001.tif]
