# Supplementary material for: Harnessing the Medicaid Analytic eXtract (MAX) to Evaluate Medications in Pregnancy: Design Considerations
Source: PLoS One. 2013 Jun 26;8(6):e67405. doi: 10.1371/journal.pone.0067405 (PMC3693950; doi:10.1371/journal.pone.0067405)
Supplement: File S1 — Supporting Information for MAX Pregnancy Cohort. Table S1: Delivery-related procedure codes used to identify inpatient and outpatient deliveries from the Medicaid Analytic eXtract, 2000-2007. Table S2: Elements of the Case Number used for linkage, enrollment type exclusions, number of pregnancies, percentage of cohort, and percentage of pregnancies with at least one prescription medication dispensed during pregnancy by state; Medicaid Analytic eXtract, 2000–2007. Table S3: Demographic characteristics on the delivery date among women in the base cohort and women in the linked pre-eligibility cohort; Medicaid Analytic eXtract, 2000–2007. Table S4. The total number of pregnancies and the percentage of pregnancies that have more than one infant MSIS_ID from the infant outcomes cohort, and the number of pregnancies with one infant MSIS_ID from the infant outcomes cohort and the percentage of pregnancies in which woman-infant pairs did not share the same zip code among pregnancies with one infant MSIS_ID from the infant outcomes cohort by state; Medicaid Analytic eXtract, 2000-2007. (PDF) [file pone.0067405.s002.pdf]

Supporting Information for MAX Pregnancy Cohort

Table S1. Delivery-related procedure codes used to identify inpatient and outpatient deliveries from the Medicaid Analytic eXtract, 2000-2007.

Code	Description
**CPT-4**	
01960	Anesthesia for vaginal delivery only
01961	Anesthesia for cesarean delivery only
01962	Anesthesia for urgent hysterectomy following delivery
01963	Anesthesia for cesarean hysterectomy w/o any labor analgesia/anesthesia care
01967	Neuraxial labor analgesia/anesthesia, planned vaginal delivery
01968	Anesthesia for cesarean delivery following neuraxial labor analgesia/anesthesia
01969	Anesthesia for cesarean hysterectomy following neuraxial labor
59050	Fetal monitoring in labor, physician w/written report
59051	Fetal monitoring in labor, physician w/written report; interpretation only
59400	Routine obstetric care, antepartum care, vaginal delivery, & postpartum care
59409	Vaginal delivery only (w/wo episiotomy &/or forceps)
59410	Vaginal delivery only (w/wo episiotomy &/or forceps); w/postpartum care
59412	External cephalic version, w/wo tocolysis
59414	Delivery, placenta (separate procedure)
59430	Postpartum care only (separate procedure)
59510	Routine obstetric care w/antepartum care, cesarean delivery, & postpartum care
59514	Cesarean delivery only
59515	Cesarean delivery only; w/postpartum care
59525	Subtotal/total hysterectomy after cesarean delivery
59610	Routine obstetric care, vaginal delivery, w/ antepartum, postpartum care, previous c-section
59612	Vaginal delivery only, previous cesarean delivery
59614	Vaginal delivery only, previous cesarean delivery; w/postpartum care
59618	Routine obstetric care, ante/postpartum, cesarean delivery after failed vaginal delivery, previous cesarean delivery
59620	Cesarean delivery, after failed vaginal delivery, previous cesarean delivery
59622	Cesarean delivery, after failed vaginal delivery, previous cesarean delivery; w/postpartum care
99436	Attendance at delivery, at request of delivering physician, & stabilization of newborn
99440	Newborn resuscitation
**ICD-9**	
72	Forceps, vacuum, and breech delivery
72.0	Low forceps operation
72.1	Low forceps operation with episiotomy
72.2	Mid forceps operation
72.21	Mid forceps operation with episiotomy
72.29	Other mid forceps operation
72.3	High forceps operation
72.31	High forceps operation with episiotomy

72.39	Other high forceps operation
72.4	Forceps rotation of fetal head
72.5	Breech extraction
72.51	Partial breech extraction with forceps to aftercoming head
72.52	Other partial breech extraction
72.53	Total breech extraction with forceps to aftercoming head
72.54	Other total breech extraction
72.6	Forceps application to aftercoming head
72.7	Vacuum extraction
72.71	Vacuum extraction with episiotomy
72.79	Other vacuum extraction
72.8	Other specified instrumental delivery
72.9	Unspecified instrumental delivery
73	Other procedures inducing or assisting delivery
73.0	Artificial rupture of membranes
73.01	Induction of labor by artificial rupture of membranes
73.09	Other artificial rupture of membranes
73.1	Other surgical induction of labor
73.2	Internal and combined version and extraction
73.21	Internal and combined version without extraction
73.22	Internal and combined version with extraction
73.3	Failed forceps
73.4	Medical induction of labor
73.5	Manually assisted delivery
73.51	Manual rotation of fetal head
73.59	Other manually assisted delivery
73.6	Episiotomy
73.8	Operations on fetus to facilitate delivery
73.9	Other operations assisting delivery
73.91	External version
73.92	Replacement of prolapsed umbilical cord
73.93	Incision of cervix to assist delivery
73.94	Pubiotomy to assist delivery
73.99	Other operations assisting delivery
74	Cesarean section and removal of fetus
74.0	Classical cesarean section
74.1	Low cervical cesarean section
74.2	Extraperitoneal cesarean section
74.4	Cesarean section of other specified type
74.9	Cesarean section of unspecified type
74.91	Hysterotomy to terminate pregnancy
74.99	Other cesarean section of unspecified type
75.4	Manual removal of retained placenta

Abbreviations: CPT-4, *Current Procedural Terminology*, Fourth Edition; ICD-9, *International Classification of Diseases*, Ninth Revision.

Table S2. Elements of the Case Number used for linkage, enrollment type exclusions, number of pregnancies, percentage of cohort, and percentage of pregnancies with at least one prescription medication dispensed during pregnancy by state; Medicaid Analytic eXtract, 2000-2007.

State	Case Number Elements	Enrollment Types Excluded	N Pregnancies	% of Cohort	% with ≥1 Medication During Pregnancy
AK	Full	None	652	0.1	89.0
AL	Full	Traditional	12300	1.0	95.7
AR	First 7 Digits	None	2610	0.2	90.2
CA	Full	None	257148	20.6	87.6
CO	First 8 Digits	Capitated	9113	0.7	89.9
CT	No Linkage	-	0	0	0
DC	Full	Capitated	533	0.0	91.0
DE	Full	Traditional	8380	0.7	94.0
FL	Full	Capitated	26582	2.1	92.8
GA	Full	None	32221	2.6	93.5
HI	Full	None	9619	0.8	87.2
IA	First 9 Digits	None	16372	1.3	94.9
ID	Full	None	4086	0.3	95.1
IL	Full	None	101031	8.1	92.6
IN	Full	None	46628	3.7	86.8
KS	Full	None	13538	1.1	92.7
KY	Full	None	28360	2.3	94.5
LA	Full	None	27514	2.2	92.0
MA	Full	Capitated	20267	1.6	95.2
MD	Full	Traditional	19459	1.6	91.6
ME	Full	None	4554	0.4	95.1
MI	Full	Exclude All	0	0	0
MN	Full	None	41357	3.3	92.0
MO	Full	None	47860	3.8	93.8
MS	Full	None	30087	2.4	94.4
MT	No Linkage	-	0	0	0
NC	Full	Capitated	25042	2.0	95.3
ND	Full	None	3655	0.3	87.4
NE	Full	None	5599	0.4	96.4
NH	Full	None	3193	0.3	92.5
NJ	First 10 Digits	None	29330	2.3	92.8
NM	Full	None	17549	1.4	90.7
NV	Full	Capitated	1381	0.1	89.7
NY	Full	None	94713	7.6	89.6
OH	Full	Capitated	54968	4.4	96.9
OK	Full	None	24206	1.9	91.2
OR	Full	None	15753	1.3	41.6
PA	Full	Capitated	14677	1.2	95.6
RI	Full	None	5485	0.4	94.6
SC	Full	Capitated	9439	0.8	96.0
SD	Full	None	5139	0.4	65.3
TN	Full	None	71164	5.7	96.0

TX	Full	Capitated	15198	1.2	95.8
UT	Full	None	5157	0.4	93.8
VA	Full	None	18991	1.5	84.5
VT	Full	None	6334	0.5	90.8
WA	First 10 Digits	Capitated	4472	0.4	95.8
WI	Full	None	50216	4.0	93.4
WV	Full	Capitated	5781	0.5	96.1
WY	Full	None	1162	0.1	89.9
**Total**	-	-	**1248875**	**100.0**	**91.0**

Table S3**.** Demographic characteristics on the delivery date among women in the base cohort and women in the linked pre-eligibility cohort; Medicaid Analytic eXtract, 2000-2007.

Characteristic	Base Cohort N=1,248,875		Linked Pre-Eligibility Cohort N=6,107,572	
Age, Years (Median, IQ Range)	23	7	23	7
Race (N, %)				
White	509122	40.8	2897648	47.4
Black	414192	33.2	1551463	25.4
Hispanic	230844	18.5	1154984	18.9
Asian or Pacific Islander	42287	3.4	194288	3.2
Other	25709	2.1	130013	2.1
Unknown	26721	2.1	179176	2.9
Eligibility (N, %)				
Disabled	38439	3.1	59310	1.0
Child <21	196038	15.7	640665	10.5
Adult*	1014398	81.2	5407213	88.5
Unknown or Not Eligible	0	0	384	0

*Adult includes women with dependent children and women who became eligible for Medicaid because of pregnancy.

Table S4. The total number of pregnancies and the percentage of pregnancies that have more than one infant MSIS_ID from the infant outcomes cohort, and the number of pregnancies with one infant MSIS_ID from the infant outcomes cohort and the percentage of pregnancies in which woman-infant pairs did not share the same zip code among pregnancies with one infant MSIS_ID from the infant outcomes cohort by state; Medicaid Analytic eXtract, 2000-2007.

State	N Pregnancies Total	% of Pregnancies with >1 Infant MSIS_ID	N Pregnancies with 1 Infant MSIS_ID	% of Pregnancies not Matching on Zip Code
AK	634	1.4	625	7.4
AL	11617	5.2	11011	8.5
AR	2170	2.2	2122	10.2
CA	209599	8.2	192379	3.4
CO	9024	1.3	8903	2.4
CT	0	0	0	0
DC	491	1.2	485	3.1
DE	8363	1.6	8226	0.6
FL	25476	2.7	24796	7.2
GA	31627	10.7	28246	5.6
HI	9475	1.3	9351	3.3
IA	16253	1.4	16029	3.9
ID	4027	1.6	3962	6.1
IL	99787	1.4	98346	12.2
IN	46464	1.5	45752	2.3
KS	13257	1.8	13018	14.2
KY	27389	6.5	25613	10.2
LA	25136	26.7	18429	4.1
MA	19944	5.2	18917	2.0
MD	19218	1.8	18865	10.5
ME	4381	1.7	4308	1.2
MI	0	0	0	0
MN	40834	10.9	36391	1.6
MO	47415	1.9	46529	3.7
MS	28438	15.5	24023	9.5
MT	0	0	0	0
NC	24800	1.8	24351	5.7
ND	3619	1.4	3569	3.4
NE	5513	1.1	5451	9.8
NH	3154	1.4	3109	1.0
NJ	27407	21.1	21634	0.5
NM	17022	6.2	15965	5.7
NV	1243	13.5	1075	2.6
NY	92946	2.9	90209	0.4
OH	54339	1.7	53424	2.9
OK	23351	4.7	22249	13.5

OR	15607	1.8	15334	13.2
PA	14640	1.4	14440	1.9
RI	5408	1.7	5316	1.0
SC	9395	2.7	9142	0.7
SD	5056	1.7	4970	2.9
TN	64833	1.7	63716	4.8
TX	14983	1.7	14727	3.5
UT	5120	1.3	5055	5.8
VA	18288	2.4	17857	3.5
VT	6116	3.6	5897	0.6
WA	3798	1.3	3747	2.3
WI	49072	19.2	39636	3.3
WV	5406	19.3	4365	4.7
WY	1145	3.8	1101	4.1
**Total**	**1173280**	**6.0**	**1102665**	**4.9**
